# Raw meat-based diet for pets: a neglected source of human exposure to *Salmonella* and pathogenic *Escherichia coli* clones carrying *mcr*, Portugal, September 2019 to January 2020

**DOI:** 10.2807/1560-7917.ES.2024.29.18.2300561

**Published:** 2024-05-02

**Authors:** Marisa Ribeiro-Almeida, Joana Mourão, Mafalda Magalhães, Ana R Freitas, Carla Novais, Luísa Peixe, Patrícia Antunes

**Affiliations:** 1 UCIBIO – Applied Molecular Biosciences Unit, Laboratory of Microbiology, Department of Biological Sciences, Faculty of Pharmacy, University of Porto, Porto, Portugal; 2 Associate Laboratory i4HB - Institute for Health and Bioeconomy, Faculty of Pharmacy, University of Porto, Porto, Portugal; 3 School of Medicine and Biomedical Sciences, University of Porto (ICBAS-UP), Porto, Portugal; 4 Centre for Innovative Biomedicine and Biotechnology (CIBB), University of Coimbra, Coimbra, Portugal; 5 Faculty of Nutrition and Food Sciences, University of Porto, Porto, Portugal; 6 1H-TOXRUN, One Health Toxicology Research Unit, University Institute of Health Sciences (CESPU-CRL), Gandra, Portugal

**Keywords:** pet food, raw meat, *Salmonella*, *E. coli*, epidemic clones, *mcr* genes, multidrug resistance, food safety, antimicrobial resistance

## Abstract

**Background:**

The pet industry is expanding worldwide, particularly raw meat-based diets (RMBDs). There are concerns regarding the safety of RMBDs, especially their potential to spread clinically relevant antibiotic-resistant bacteria or zoonotic pathogens.

**Aim:**

We aimed to investigate whether dog food, including RMBD, commercially available in Portugal can be a source of *Salmonella* and/or other *Enterobacteriaceae* strains resistant to last-line antibiotics such as colistin.

**Methods:**

Fifty-five samples from 25 brands (21 international ones) of various dog food types from 12 suppliers were screened by standard cultural methods between September 2019 and January 2020. Isolates were characterised by phenotypic and genotypic methods, including whole genome sequencing and comparative genomics.

**Results:**

Only RMBD batches were contaminated, with 10 of 14 containing polyclonal multidrug-resistant (MDR) *Escherichia coli* and one MDR *Salmonella*. One turkey-based sample contained MDR *Salmonella* serotype 1,4,[5],12:i:- ST34/cgST142761 with similarity to human clinical isolates occurring worldwide. This *Salmonella* exhibited typical antibiotic resistance (*bla*_TEM_ + *strA-strB* + *sul2* + *tet(B)*) and metal tolerance profiles (*pco* + *sil* + *ars*) associated with the European epidemic clone. Two samples (turkey/veal) carried globally dispersed MDR *E. coli* (ST3997-complexST10/cgST95899 and ST297/cgST138377) with colistin resistance (minimum inhibitory concentration: 4 mg/L) and *mcr-1* gene on IncX4 plasmids, which were identical to other IncX4 circulating worldwide.

**Conclusion:**

Some RMBDs from European brands available in Portugal can be a vehicle for clinically relevant MDR *Salmonella* and pathogenic *E. coli* clones carrying genes encoding resistance to the last-line antibiotic colistin. Proactive actions within the One Health context, spanning regulatory, pet-food industry and consumer levels, are needed to mitigate these public health risks.

**Key public health message**

**What did you want to address in this study and why?**

Raw meat-based diets (RMBDs) are increasingly popular among pet owners. However, the potential role of RMBDs has been neglected as a new source of bacteria resistant to last-resort antibiotics which could affect people co-living with pets. We wanted to analyse if dog food from brands sold in the European Union represents a possible source of *Salmonella* or other bacteria resistant to the important antibiotic colistin.

**What have we learned from this study?**

Conventionally processed pet food is a safer option than RMBDs. This is because RMBDs of European brands can carry multidrug-resistant bacteria, including globally disseminated pathogenic *Salmonella* and *E. coli* harbouring genes encoding resistance to colistin, an antibiotic critically important for human medicine. These hazards are frequent in food-animal production and are causing infections in humans worldwide.

**What are the implications of your findings for public health?**

The detection in RMBDs of a predominant pandemic *Salmonella* clone and pathogenic *E. coli* carrying mobile colistin resistance genes may pose a potential risk of human exposure. This can occur through handling of pet food and/or environmental release by pets. These findings indicate a need for proactive actions involving the pet industry, food safety agencies, and pet owners to mitigate risks for public health.

## Introduction

The pet industry has evolved in recent decades due to increasing pet populations, stronger human–pet bonds and demand for high-quality pet food products [1,2]. Processed pet food manufactured with various processing methods (e.g. grinding, cooking, extrusion and dehydration) has traditionally been considered microbiological safe and nutritionally suitable for feeding pets [1,3]. However, since some pet owners consider unprocessed food healthier, raw meat-based diets (RMBDs) for dogs have gained popularity [1,2,4]. The RMBDs are mainly composed of uncooked or minimally processed meat, bones and organs, with freezing as the primary treatment, and are considered to be more natural than conventional processed pet food [1,5]. Nevertheless, the scientific evidence supporting RMBD benefits is scarce, and many veterinary professional organisations (e.g. the World Small Animal Veterinary Association) and international public health agencies (e.g. the United States (US) Centers for Disease Control and Prevention (CDC)) view them as potential health hazards for both animals and humans [1,5]; awareness of this issue appears less evident in Europe [6]. The safety concerns associated with RMBDs are related to the potential contamination of raw ingredients with zoonotic pathogenic bacteria and parasites [1,3,4]. Such contamination could lead to the spread of these pathogens to both pets and humans cohabitating with pets, through direct contact with the pet or its feed, or indirectly through contact with contaminated household surfaces or hands during feed preparation.

In the European Union (EU), legal requirements for the use of animal by-products and derived products not intended for human consumption are established, including those to produce processed or raw pet food, helping to ensure microbiological safety [7]. Nevertheless, since 2020, there have been more than 20 notifications or recalls of pet food and RMBD in the EU due to the detection of zoonotic pathogens, particularly *Salmonella* and pathogenic *Escherichia coli* [8], and also cases of human infections with *Salmonella* and Shiga toxin-producing *E. coli* (STEC) linked to exposure to RMBDs [9-11]. Several studies have also established a correlation between the microbiota of pets and their owners, including the presence of antibiotic-resistant strains, with pet food as a potential source [12,13]. However, certain antibiotic-resistant bacteria and genes of public health concern, such as the *mcr* gene conferring resistance to the last-line antibiotic colistin, have not been extensively studied in pet food and RMBDs [1,14-16]. Consequently, these antibiotic-resistant strains and genes have not been recognised as notable food safety issues in the context of the pet food industry [6]. To address this knowledge gap, we aimed to investigate the occurrence of and further characterise *Salmonella* and other *Enterobacteriaceae* resistant to critical antibiotics, such as colistin, in dog food, including RMBDs, that is available in stores in Portugal to investigate if they represent a possible source of these hazards to public health. 

## Methods

### Sampling strategy

We visited physical locations such as major supermarkets and pet stores in the Porto metropolitan area and conducted an online search to gather information on the primary canine food types and brands commercially accessible in Portugal. Over 5 months (September 2019 to January 2020), 55 dog food samples (22 wet, 14 raw-frozen, eight dry, seven treats and four semi-moist), corresponding to 50 different dog food items (four food types were acquired 2–3 times) and 25 brands commercialised in Portugal, were collected from 12 retail stores (eight supermarkets, three specialised stores and one veterinary clinic) in the Porto region; further details about the samples are appended in Supplementary Table S1. Most samples were obtained from brands marketed globally, including in the EU (21/25). The 14 raw-frozen dog food samples were a combination of fruits, vegetables and different types of meat (muscle/viscera). They were categorised into two groups based on the main meat type: poultry (n = 8; chicken, turkey, duck, goose) and ruminant (n = 6; veal, steer, deer) samples. The RMBDs were from the two international brands available in Portuguese stores (brand A types produced in the EU and brand B in the United Kingdom (UK)). The samples were processed according to their type, as previously described [17]. For the pre-enrichment step at 37 °C for 16–18 h, 25 g of each sample were homogenised (2 min in a Stomacher blender) with 1:10 buffered peptone water (BPW).

### Detection of non-typhoidal *Salmonella*

*Salmonella* detection was performed using the International Standard Organisation [18] method for foodstuffs. Briefly, after the pre-enrichment, 0.1 mL and 1 mL of the BPW were transferred to Rappaport–Vassiliadis medium with Soya (RVS) and Muller–Kauffmann tetrathionate-novobiocin (MKTTn) broths, respectively, for selective enrichment (RVS at 41.5 °C for 24 h and MKTTn at 34–38 °C for 24 h). These broths were then streak-plated on xylose lysine deoxycholate agar and CHROMagar *Salmonella* Plus. Presumptive *Salmonella* colonies recovered from both selective agar plates (up to five colonies per plate) were confirmed by biochemical tests (e.g. API-20 E, bioMérieux, Marcy l’Etoile, France), by agglutination with *Salmonella* O poly antisera and serogroup-specific antisera (Becton Dickinson, New Jersey, US) and by PCR for *invA* gene detection and *Salmonella* serotypes of particular concern in the EU (Enteritidis, Typhimurium and 1,4,[5],12:i:-) [19].

### Screening of *mcr*-carrying *Enterobacteriaceae*

After BPW enrichment, 100 µL and 10 µL were spread on Tryptone Bile X-glucuronide agar plates (TBX) and Simmons citrate agar + inositol (SCAi) with and without colistin (3.5 mg/L) and incubated (TBX at 37 °C for 24 h; SCAi at 37 °C for 48 h) for *E. coli* and *Klebsiella* spp. detection, respectively. From each plate, between one and five colonies of each morphotype were spread on a CLED medium for further identification by matrix-assisted laser desorption-ionisation-time of flight mass spectrometry (MALDI-TOF VITEK MS, bioMérieux) and standard PCRs for *E. coli* and *K. pneumoniae* [20]. Colistin resistance genes (*mcr-1* to *mcr-5* and *mcr-6* to *mcr-9*) were identified in *E. coli*, *K. pneumoniae* and *S. enterica* isolates using a multiplex PCR published previously [21]. Amplified simplex PCR products were purified using the NZYGelpure kit (NZYTech, Lisbon, Portugal) and sequenced at Eurofins Genomics (Konstanz, Germany).

### Phenotypic and genotypic characterisation of *Enterobacteriaceae*

We used disk diffusion to test susceptibility to the following antibiotics: amoxicillin 10 µg, amoxicillin/clavulanic acid 30 µg, cefepime 30 µg, cefoxitin 30 μg, ceftazidime 30 μg, cefotaxime 30 μg, meropenem 10 µg, ciprofloxacin 5 μg, pefloxacin 5 μg (only for *Salmonella*) nalidixic acid 30 µg, gentamicin 10 μg, streptomycin 10 µg, kanamycin 30 µg, tobramycin 10 μg, chloramphenicol 30 μg, tetracycline 30 μg, sulfonamides 300 µg, trimethoprim 5 µg and fosfomycin 200 µg (only for *E. coli*). Colistin minimum inhibitory concentration (MIC) was determined by the European Committee on Antimicrobial Susceptibility Testing (EUCAST) reference cation-adjusted Mueller–Hinton broth microdilution method [22]. *Escherichia coli* ATCC 25922 served as the control. Interpretation followed the EUCAST guidelines [23], and for nalidixic acid and tetracycline, we used the Clinical and Laboratory Standards Institute clinical breakpoints [24]. Multidrug resistance (MDR) was defined as resistance to antibiotics from three or more different families. Phylogenetic groups (PhG) of *E. coli* were determined using a standard multiplex PCR [20]. In addition, Shiga toxin-producing *E. coli* (STEC) was assessed by PCR for *stx1* and *stx2* virulence genes [25].

### Whole genome sequencing for characterisation of *Salmonella* and *mcr-1*-carrying *Escherichia coli*

We selected one isolate per sample of *Salmonella* and *mcr-1*-positive *Enterobacteriaceae* for WGS. We extracted DNA using the Wizard Genomic DNA purification kit (Promega Corporation, Madison, US) and measured its concentration with a Qubit 3.0 Fluorometer (Invitrogen, Thermo Fisher Scientific, Massachusetts, US). The HiSeq (2 × 151 bp) Illumina platform (Illumina, San Diego, US) was used for sequencing at Eurofins Genomics. Raw reads quality was assessed with FastQC v0.11.9 (http://www.bioinformatics.babraham.ac.uk/projects/fastqc) using default parameters. High-quality reads were then de novo assembled using SPAdes v3.15.5 (https://github.com/ablab/spades) within Unicycler v0.5.0 (https://github.com/rrwick/Unicycler). Assembly quality and completeness were assessed with QUAST v5.0.2 (https://quast.sourceforge.net) and BUSCO v5.0.0 (https://github.com/WenchaoLin/BUSCO-Mod), respectively. Draft genomes were annotated on the RAST server (https://rast.nmpdr.org). For metal tolerance genes search (*arsRD2A2BCA1D1*-*arsR1HD1A1A2CBA3D2R2*, *pcoGE1ABCDRSE2*, *silESRCFBAGP*, *terFEDCBAZ*-*terY3Y2XY1W* and *merEDACPTR*), we used ABRicate v1.0.1 (https://github.com/tseemann/abricate) with an in-house database. We used tools from the Centre for Genomic and Epidemiology (http://www.genomicepidemiology.org) to evaluate *E. coli* and *Salmonella* antibiotic resistance genes (ResFinder v4.1, https://cge.food.dtu.dk/services/ResFinder) or known mutations (PointFinder v4.1, https://bitbucket.org/genomicepidemiology/pointfinder_db.git), virulence genes (only for *E. coli*, VirulenceFinder v2.0, https://cge.food.dtu.dk/services/VirulenceFinder), plasmid replicons (PlasmidFinder v2.1, https://cge.food.dtu.dk/services/PlasmidFinder), plasmid typing (pMLST v2.0, https://cge.food.dtu.dk/services/pMLST) and Multilocus Sequence Typing (MLST v2.0, https://cge.food.dtu.dk/services/MLST). *Salmonella* serotypes were confirmed with the *Salmonella* In Silico Typing Resource (SISTR) (https://github.com/phac-nml/sistr_cmd) and *E. coli* PhGs were validated using ClermontTyper (http://clermontyping.iame-research.center).

For confirmation of *mcr-1* gene location and hypothetical plasmid reconstructions, we used the MOB-recon tool v3.1.0 from the MOB-suite package (https://github.com/phac-nml/mob-suite). If the *mcr*-1 gene was identified in a plasmid by MOB-recon or on the same contig as the replicon, it was considered plasmid-associated. The PLSDB-plasmid database (https://ccb-microbe.cs.uni-saarland.de/plsdb) was used for comparative genomic analysis. Alignment of *mcr-1*-carrying plasmids with closely related IncX4 ones was conducted using the BRIG tool (v0.95) (https://github.com/happykhan/BRIG).

### Comparative genomic analysis of *Salmonella* and *mcr-1*-carrying *Escherichia coli*

We conducted a comparative genomic analysis using core-genome MLST (cgMLST) between our isolates and genomes queried from Enterobase as well as the hierarchical clustering of cgMLST (HierCC) (https://enterobase.warwick.ac.uk). These strains were used to develop a minimum-spanning tree using GrapeTree (https://achtman-lab.github.io/GrapeTree/MSTree_holder.html) and MSTreeV2. Metadata of the included *Salmonella* and *E. coli* isolates were retrieved from Enterobase (isolate name, cgST, country, year, source). In addition, we conducted a search of antibiotic resistance, metal tolerance and virulence genes as described in the previous section.

### Statistical analysis

Occurrence rates and antibiotic-resistant *E. coli* variations across food types were assessed using Fisher’s exact test (α = 0.05). The 95% confidence intervals (CI) for proportions were calculated using Wilson CI. Both analyses were computed using Prism v 9.1.1 (GraphPad, Boston, Massachusetts, US).

## Results

### Detection and characterisation of *Salmonella*

In our study of 55 pet food samples (41 processed and 14 raw), only raw samples tested positive for Gram-negative bacteria, including the zoonotic pathogen *Salmonella*, along with bioindicators *E. coli* and *Klebsiella pneumoniae* (Table 1). We detected *Salmonella* in one of the raw samples (7%; 95% CI: 1.3–31.5; n = 1/14), a raw-frozen batch (EU, brand A), predominantly containing turkey (Table 1). 

**Table 1:** Distribution of *Salmonella enterica*, *Escherichia coli* and *Klebsiella pneumoniae* among samples of commercially available raw-frozen dog food, Portugal, September 2019–January 2020 (n = 14)

Food type	Sample number	Brand^a^	Main ingredients^b^ (% of the most prevalent ingredients)	Collection date	Bacteria detected		
					*S. enterica*	*E. coli* (*mcr-1*)	*K. pneumoniae*
Ruminant-based	6	A	**Veal** (59%); salmon (20%); vegetables; salmon oil	Sept 2019	−	+	−
	46			Nov 2019	−	+	−
	53			Jan 2020	−	+	−
	18	A	**Steer** (79%); vegetables; salmon oil	Oct 2019	−	+	+
	45			Nov 2019	−	+	−
	26	B	**Deer** (80%); vegetables; fruit	Nov 2019	−	+ (*mcr-1*)	−
Poultry-based	15	A	**Chicken** (60%); lamb (19%); vegetables	Oct 2019	−	+	−
	16	A	**Chicken** (60%); veal (19%); vegetables	Oct 2019	−	+	+
	55			Jan 2020	−	+	−
	17	A	**Turkey** (60%); lamb (20%); vegetables	Oct 2019	−	+	−
	54			Jan 2020	+	+	−
	25	B	**Duck** (80%); vegetables; fruit	Nov 2019	−	+	+
	51	B	**Turkey** (50%); goose (30%); vegetables; fruit	Jan 2020	−	+	+
	52	B	**Turkey** (40%); salmon (20%); white fish (20%); vegetables	Jan 2020	−	+ (*mcr-1*)	−

−: not detected; +: detected.

^a^ Raw food samples were from either of two international brands distributed worldwide, here randomly designated as A and B.

^b^ The most prevalent ingredient in each food type is shown in bold.

Six *Salmonella* isolates, all identified as *Salmonella enterica* serotype 1,4,[5],12:i:- (Typhimurium monophasic variant) were successfully isolated from the same sample. They exhibited the MDR ASSuT profile conferring resistance to amoxicillin (A, encoded by the *bla*_TEM_ gene), streptomycin (S, *strA-strB*), sulphonamides (Su, *sul2*), and tetracycline (T, *tet(B)*). In addition, they had an integrative and conjugative element (ICE) with copper/silver (*pco, sil*) and arsenic (*ars*) tolerance clusters/operons (Table 2), typical of the widespread clinically relevant ‘European clone’ [26].

**Table 2:** Genomic characterisation of *Salmonella enterica*, and the *mcr*-1-carrying *Escherichia coli* isolates recovered from samples of commercially available raw-frozen dog food, Portugal, September 2019–January 2020 (n = 3)

Isolate name	Species	Brand (main ingredient)	Serotype	PhG MLST cgMLST^a^	Antimicrobial resistance genes; chromosomal mutations	*mcr-1* location PL-Inc (kb)	Virulence genes^b^	Acquired metal tolerance genes
R54_S100	*Salmonella enterica*	A (turkey)	1,4,[5],12:i:-	NA ST34 cgST142761	*bla*_TEM-1B,_ *aac(6')-Iaa*, *str*A, *str*B, *sul*2, *tet(*B)	NA	NS	*sil*ERSCFBAGP, *pco*GE1ABCDRSE2, *ars*RD2A2BCA1D1, *mer*EDACPTR
R26_1_100	*Escherichia coli*	B (deer)	O153:H37	B1 ST297 cgST193137	*bla*_TEM-1B_, *bla*_CARB-2_, *aadA*1, *aadA*2, *cmlA*1, *mcr-*1.1, *sul*3, *dfrA*16; *gyrA* p.S83L, *gyrA* p.D87N, *parC* p.S80I	PL-X4 (33)	*gad, lpfA,* ***ompT***, *ter*	*ars*R1BCR2
R52_8	*Escherichia coli*	B (turkey)	O101:H37	A ST3997 cgST193139	*bla*_TEM-1B,_ *mcr-*1.1, *str*A, *str*B, *tet(*B), *mdf*(A); *gyrA* p.S83L, *gyrA* p.D87N, *parC* p.S80I	PL-X4 (33)	*cma,* ***cvaC***, ***etsC***, *gad,* ***hlyF***, ***hra***, ***iroN***, ***iss***, ***ompT***, *terC,* ***traT***	*ars*R1BC

APEC: avian pathogenic *Escherichia coli*; cgMLST: core genome multilocus sequence typing; ExPEC: extraintestinal pathogenic *Escherichia coli*; MLST: multilocus sequence typing; NA: not applicable; NS: not searched; PhG: *E. coli* phylogenetic group; PL: plasmid.

^a^ cgMLST obtained from Enterobase (https://enterobase.warwick.ac.uk) for both species.

^b^ The virulence genes associated with ExPEC are highlighted in bold, while those associated with APEC are underlined; genes that belong to both are indicated in bold and underline.

**Figure 1:**
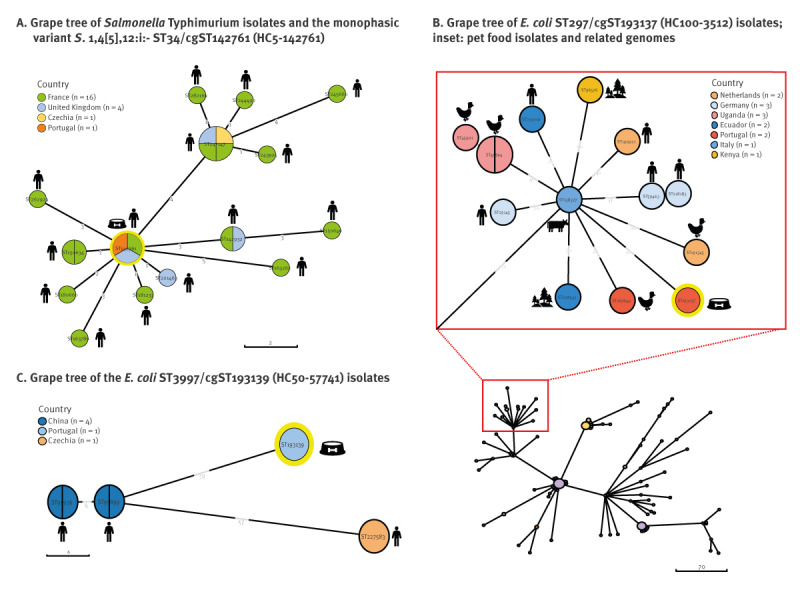
Phylogenetic trees of *Salmonella* Typhimurium and *Escherichia coli* isolates from raw pet food samples, Portugal, 2020 (n = 3) and related available genomes on EnteroBase up to 8 June 2023 (n = 22 *Salmonella* Typhimurium, n = 20 *E. coli*)

The *S*. 1,4,[5],12:i:- strain belonged to sequence type ST34 (Table 2), which is commonly observed in Europe, as evidenced by the data available on EnteroBase (https://enterobase.warwick.ac.uk/species/index/senterica). Using the EnteroBase cgMLST scheme, the pet food isolate was classified as cgST142761, which grouped into a distinct cluster (Hierarchical Clustering-HierCC HC5–142761 group) among the globally dispersed ST34 clone (Figure 1A). This isolate exhibited a close genetic relationship (HC5) with 21 isolates of human origin (Czechia, France and UK; 2018–2022), with no apparent clustering based on European geographical location (Figure 1A); Supplementary Table S2 contains further strain details.

The core genome minimum spanning tree was created within the EnteroBase pipeline using the MSTreeV2 algorithm and GrapeTree tool. The cgST is indicated in each node. The yellow circle highlights the pet food isolate of each Grape tree. For the geographical analysis, the cgST was annotated using the country data (the number of genomes by country is indicated within parentheses). The scale bar corresponds to the number of cgMLST allelic differences.

### Detection and characterisation of colistin-resistant Enterobacteriaceae

We detected *E. coli* (n = 59 isolates; none STEC) in all raw-frozen food samples (100%; 95% CI: 79–100; n = 14/14). Four of them were also contaminated with *K. pneumoniae* (n = 5 isolates) (Table 1). A considerable percentage (71%; 95% CI: 45–88; n = 10/14) of the samples carried MDR *E. coli* isolates, independently of the main ingredients and PhGs (Table 3). Antibiotic resistance rates were similar between samples with poultry or ruminant-based ingredients (p > 0.05). More than half of the samples contained at least one *E. coli* with resistance to amoxicillin (79%; 95% CI: 52–92; n = 11/14 samples), ciprofloxacin (50%; 95% CI: 27–73; n = 7/14), nalidixic acid (57%; 95% CI: 33–79; n = 8/14), streptomycin (71%; 95% CI: 45–88; n = 10/14), tetracycline, sulphonamides or trimethoprim (64%; 95% CI: 39–84; n = 9/14 each); For further antibiotic resistance details, we refer to Supplementary Figure S1.

**Table 3:** Characteristics of *Escherichia coli* recovered from samples of commercially available raw-frozen dog food, Portugal, September 2019–January 2020 (n = 59 isolates)

Food type	Sample ID (brand)	Main ingredients^a^ (% of the most prevalent ingredients)	PhG (number of isolates)	Antibiotic resistance profile^b^	*mcr-1* gene detection	MDR (sample ID)^c^
Ruminant-based	6, 46, 53 (A)	**Veal** (59%); salmon (20%); vegetables; salmon oil	A (2)	AML (STR, SUL, TMP)	−	**+** (53)
			B1 (8)	−	−	−
			B2 (2)	−	−	−
			D (1)	−	−	−
	18, 45 (A)	**Steer** (79%); vegetables; salmon oil	A (2)	(AML, STR, CHL, TET, SUL, TMP)	−	**+** (45)
			B1 (4)	(AML, STR, NAL, TET)	−	**+** (18)
	26 (B)	**Deer** (80%); vegetables; fruit	A (2)	TET	−	−
			B1 (4)	(AML, AMC, CIP, NAL, STR, CHL, COL, TET, SUL, TRP)	**+**	**+**
Poultry-based	15 (A)	**Chicken** (60%); lamb (19%); vegetables	B1 (4)	AML, TET, SUL, TMP (AMC, CHL, CIP, NAL, STR, KAN)	−	**+**
	16, 55 (A)	**Chicken** (60%); veal (19%); vegetables	A (10)	(AML, CIP, NAL, STR, TET, SUL, TMP)	−	**+**
			C (2)	AML, CIP, NAL, STR, TET, SUL, TMP (FOX)	−	**+** (55)
	17, 54 (A)	**Turkey** (60%); lamb (20%); vegetables	B1 (2)	−	−	−
			D (2)	STR, TET, SUL, TMP (AML, CIP, NAL)	−	**+** (17)
	25 (B)	**Duck** (80%); vegetables; fruit	A (1)	−	−	−
			B1 (1)	−	−	−
	51 (B)	**Turkey** (50%); goose (30%); vegetables; fruit	A (1)	NAL, TET	−	−
			B1 (7)	CIP, NAL, TET (AML, GEN, STR, KAN, TOB, CHL, SUL, TRP)	−	**+**
	52 (B)	**Turkey** (40%); salmon (20%); white fish (20%); vegetables	A (3)	AML, AMC, STR, TET (CIP, NAL, KAN, GEN, COL, SUL, TRP) AML, AMC, CIP, NAL, GEN, STR, TET, SUL, TRP	**+**	**+**
			B1 (1)		−	**+**

−: not detected; +: detected; AMC: amoxicillin + clavulanic acid; AML: amoxicillin; CHL: chloramphenicol; CIP: ciprofloxacin; COL: colistin; FOX: cefoxitin; GEN: gentamicin; ID: identification number; KAN: kanamycin; MDR: multidrug resistance; NAL: nalidixic Acid; PhG: *E. coli* Phylogenetic Group; STR: streptomycin; SUL: sulphonamides; TET: tetracycline; TOB: tobramycin; TRP: trimethoprim.

^a^ The most prevalent ingredient in each food type is represented in bold.

^b^ Variable presence of antibiotic resistance is presented in brackets. (−) indicates isolates susceptible to all tested antibiotics.

^c^ The sample ID number is indicated only when more than one food sample type was analysed.

**Figure 2:**
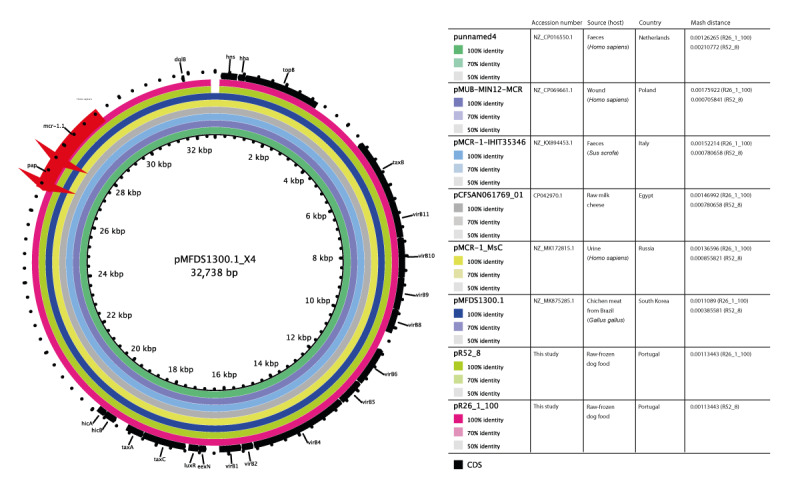
Circular maps of *mcr-1*-harbouring IncX4 plasmids in *Escherichia coli* isolated from Portuguese pet food, September 2019–January 2020 (n = 2) and closely related ones from different sources and geographical regions

Colistin-resistant *E. coli* isolates (n = 4) were present in two batches (14%; 95% CI: 4–40; n = 2/14) from the same pet food brand (UK, brand B), one with deer and the other with turkey as the main ingredient (Table 3). All isolates carried the *mcr-1* gene with MIC = 4 mg/L and were recovered in TBX medium supplemented with colistin. They were co-resistant to amoxicillin, streptomycin, ciprofloxacin, nalidixic acid, streptomycin and sulphonamides. These *mcr-1*-carrying *E. coli* isolates belonged to B1-ST297 and A-ST3997-ST10 complex-Cplx (Table 2). The ST297 (cgST193137), classified as an extraintestinal pathogenic *E. coli* (ExPEC), shared a common HC100 cluster (Hierarchical Clustering-HierCC HC100–3512 group) with 162 genomes and the lowest number of allelic differences with 13 genomes from globally dispersed sources and countries (Germany, Italy, the Netherlands, Ecuador, Kenya and Uganda; 2014–2021), including from a Portuguese poultry farm (Figure 1B); Supplementary Table S3 provides further strain details. The ST3997-ST10 Cplx (cgST193139) presented virulence genes associated with avian pathogenic *E. coli* (APEC) and shared a single cluster at the HC50 level with five isolates from humans in Europe (Czechia, 2020) and Asia (China, 2017) (Figure 1-C); for further strain details we refer to Supplementary Table S4. Whole genome sequencing revealed that in both *E. coli* strains from the same pet food brand, the *mcr-1.1* gene was located on similar IncX4 plasmids (99.89% identity). These plasmids shared a common genetic environment near the *mcr*-1 cassette, contained the *pap2* gene (membrane-associated lipid phosphatase) and lacked the ISApI1 element [16]. Comparative genomics revealed that these IncX4 plasmids were similar to others (MOB-recon; mash distance: 0.000780658–0.00126265) and are circulating among diverse hosts (humans, pig, poultry) and the environment in many different countries, including Portugal (Figure 2); Supplementary Table S5 provides further plasmid details.

Alignment of R26_1_100 of 33789 bp and R52_8 of 32534 bp (Portugal, 2020) against others. Black inner ring: plasmid used as a reference for the alignment; name and size of the reference indicated in the middle of the panel. Genes are represented by black arrows (indicating the position and direction of transcriptional open-reading frames) in the outer circle, with *mcr-1.1* and *pap* genes highlighted in red.

## Discussion

This study investigated the presence and characteristics of *Salmonella* and other *Enterobacteriaceae* in 55 dog food samples, with a focus on colistin-resistant strains. These samples comprised various types of meat and were obtained from different suppliers and international brands in Portugal. We found *Enterobacteriaceae*, including *Salmonella* and MDR isolates, only in samples from raw pet food, in contrast to a parallel study in the same samples [17], where *Enterococcus* spp. was detected across all sample types, including dry and wet. Current regulations in the EU propose counting *Enterobacteriaceae* (and including *Salmonella* detection) as a hygiene criterion for all categories of pet foods [7]. Numerous studies have demonstrated contamination levels exceeding the EU limits (i.e. *Salmonella*: absence in 25 g and *Enterobacteriaceae* < 5 × 10^3^ CFU/g) in RMBDs [4,5,14,15,27]. Our results strongly suggest that conventionally processed pet food is a safer option, emphasising the critical role of heat treatment in pet food production for effectively mitigating microbiological hazards [1,2].

Although the overall prevalence of *Salmonella* in the RMBDs samples in this study was low (one of 14 batches produced in the EU had unsatisfactory microbiological quality), other studies from Europe also detected *Salmonella*: 4% of raw pet food samples in Switzerland, 20% in the Netherlands and 71% in Italy [14,16,27]. The *Salmonella enterica* detected in this study was of the serotype 1,4,[5],12:i:- and belonged to ST34, which has emerged as the predominant pandemic genotype in recent decades, particularly in food animal production and human infections in the EU [26,28]. Their MDR features (ASSuT + ICE) may have facilitated the adaptation of this serotype to environments with extensive usage of antibiotics and heavy metals, such as pig and poultry farms [26,28,29], whose raw animal by-products are the sources of the pet food industry. Since food animals are asymptomatic carriers of *Salmonella*, these bacteria can spread easily at slaughterhouses through cross-contamination events between flocks or animal by-products or at pet food production plants, in various types of meat, animal species and geographical places of origin [6,19,30]. Notably, we showed genetic similarities between *S*. 1,4,[5],12:i:- from RMBDs and public genomes from human clinical cases from different European countries, suggesting a role of raw pet food as a potential vehicle for the transmission of this serotype considered of human health significance in the EU and carrying a MDR profile. Some studies consistently show a significant difference in *Salmonella* excretion in faeces between dogs fed with RMBDs and those fed with dry food, highlighting the microbiological risk associated with RMBDs [3,31,32]. This risk extends not only to dogs but also to pet owners handling RMBD and dog faeces, as well as to the environment, as documented by recent *Salmonella* outbreaks where WGS confirmed a connection between pets, pet food and human disease [10,11,33,34].

A high percentage of our samples carried MDR *E. coli* isolates, regardless of the raw food types tested, similar to a recent parallel study focused on MDR *Enterococcus* in dog food in Portugal [14]. Resistance to commonly used veterinary antibiotics such as β-lactams, fluoroquinolones, tetracycline and sulphonamides was especially pronounced, mirroring trends seen in other European studies on pet food samples of diverse origins [14,35]. The use of these antibiotics, particularly in poultry production, has been associated with increased *E. coli* resistance rates [29], suggesting that raw meat-based ingredients might be introducing MDR strains in pet food, which then can persist until they reach humans and their pets [32]. While the percentage of samples containing MDR and *mcr*-carrying *E. coli* isolates was relatively low, in line with findings from other studies [14,16], it underscores the importance of employing antibiotics judiciously within the livestock industry. This is needed to curb the co-selection of genes conferring resistance to colistin, a “highest priority critically important antimicrobial” for human medicine among various bacterial species [20,21]. In fact, *E. coli* ST297 (ExPEC) and ST3997-ST10 Cplx (APEC) identified in this study have been detected worldwide in various animal, food, environmental and human sources, and have been linked to numerous human infections (https://enterobase.warwick.ac.uk/species/index/ecoli), which highlights their capacity to be transmitted to humans through the food chain. In Portugal, the MDR *E. coli* ST297 lineage is predominant in many food sources [20,36] and has now been detected in raw pet food in our study. Meanwhile, in Asia, *E. coli* ST3997-ST10 Cplx isolates found in poultry, the environment and humans also carried *mcr-1* associated with diverse plasmid backgrounds [37]. Notably, both *E. coli* strains obtained in this study from the same pet food brand carried the *mcr-1.1* gene on similar IncX4 plasmids. These findings, along with the similarity of these plasmids to globally distributed ones, suggest possible cross-contamination events and/or diverse origins of pet food contamination arising from ingredients or human handling at the production plant.

In this study, RMBDs were identified as a potential vehicle of MDR zoonotic-related pathogenic bacteria, with some carrying genes such as *mcr-1* conferring resistance to last-line antibiotics. Despite the EU's efforts to reduce antibiotics such as colistin in livestock production and the successful colistin restrictions on EU farms [20,21], the introduction of colistin-resistant bacteria through imported animal by-products (e.g. from non-EU countries with different antibiotic practices and regulations), raw vegetables (common in most samples) or wildlife (e.g. deer as the main ingredient in one batch with *mcr*-carrying *E. coli*) cannot be excluded. Continuous vigilance is essential to address these potential pathways and mitigate the spread of antibiotic-resistant bacteria. Furthermore, most manufacturers do not provide information on food safety practices (e.g. handwashing, safe handling) for handling raw pet food [38], including on labels found on the raw pet food samples obtained for this study. Appropriate hygiene measures and safe handling practices should be observed when dealing with pets and raw pet food to mitigate the risk of MDR bacterial infections in humans.

Finally, we acknowledge the study's limitations. Firstly, the results should be interpreted considering our convenience-based sampling strategy, which exclusively captured dog food types and brands available on the Portuguese pet food market, primarily in four cities in the Porto metropolitan area. Consequently, the results may not be extrapolated to pet food products available from other suppliers. Secondly, the small sample size and the uneven distribution among suppliers and various canine food items may have introduced unintended selection bias. Moreover, additional studies, encompassing brands available in every region in the world, along with local risk assessment investigations, are required to discern the broader implications of pet food on public health.

## Conclusion

This study demonstrates that RMBDs from European brands available in Portugal can be a vehicle for MDR clinically-relevant *Salmonella* and *E. coli* carrying genes encoding resistance to the last-resort antibiotic colistin. Promoting awareness of potential risks linked to RMBDs and providing guidance to pet owners on proper handling and feeding practices are crucial steps in minimising potential health risks. Identifying environmental transmission routes of pathogenic and MDR bacteria to pet food and the continuous microbiological monitoring (pathogens and antibiotic-resistant bacteria/genes) of ingredients and processes used in the fast-growing pet food industry needs to be addressed in future One Health studies to mitigate public health risks.

## Supplementary Data

41000 Supplementary_Tables

## Supplementary Data

251000 Supplementary_Figure

## References

[1] Davies RH Lawes JR Wales AD . Raw diets for dogs and cats: a review, with particular reference to microbiological hazards. J Small Anim Pract. 2019;60(6):329-39. 10.1111/jsap.13000 31025713 PMC6849757

[2] Freeman LM Chandler ML Hamper BA Weeth LP . Current knowledge about the risks and benefits of raw meat-based diets for dogs and cats. J Am Vet Med Assoc. 2013;243(11):1549-58. 10.2460/javma.243.11.1549 24261804

[3] Runesvärd E Wikström C Fernström L-L Hansson I . Presence of pathogenic bacteria in faeces from dogs fed raw meat-based diets or dry kibble. Vet Rec. 2020;187(9):e71. 10.1136/vr.105644 32054718 PMC7799416

[4] Solís D Toro M Navarrete P Faúndez P Reyes-Jara A . Microbiological quality and presence of foodborne pathogens in raw and extruded canine diets and canine fecal samples. Front Vet Sci. 2022;9:799710. 10.3389/fvets.2022.799710 35923819 PMC9339799

[5] Vecchiato CG Schwaiger K Biagi G Dobenecker B . From nutritional adequacy to hygiene quality: A detailed assessment of commercial raw pet-food for dogs and cats. Animals (Basel). 2022;12(18):2395. 10.3390/ani12182395 36139257 PMC9495138

[6] European Pet Food Industry (FEDIAF). Guide to good practice for the manufacture of safe pet foods. Brussels: FEDIAF; 2018. Available from: http://europeanpetfood.org/wp-content/uploads/2022/03/FEDIAF_Safety_Guide_February_2018_online.pdf

[7] Europe Commission. Commission Regulation (EU) No 142/2011 implementing Regulation (EC) No 1069/2009 of the European Parliament and of the Council laying down health rules as regards animal by-products and derived products not intended for human consumption and implementing Council Directive 97/78/EC as regards certain samples and items exempt from veterinary checks at the border under that Directive. Official Journal of the European Union. 26.2.2011:L 54/1. Available from: http://www.eur-lex.europa.eu/LexUriServ/LexUriServ.do?uri=OJ:L:2011:054:0001:0254:EN:PDF

[8] Rapid Alert System for Food and Feed (RASFF). RASFF Window. Brussels: European Commission. [Accessed: 12 Feb 2024]. Available from: https://webgate.ec.europa.eu/rasff-window/screen/search

[9] Kaindama L Jenkins C Aird H Jorgensen F Stoker K Byrne L . A cluster of Shiga toxin-producing Escherichia coli O157:H7 highlights raw pet food as an emerging potential source of infection in humans. Epidemiol Infect. 2021;149(e124):e124. 10.1017/S0950268821001072 33955833 PMC8161292

[10] Russini V Corradini C Rasile E Terracciano G Senese M Bellagamba F A familiar outbreak of monophasic Salmonella serovar Typhimurium (ST34) involving three dogs and their owner’s children. Pathogens. 2022;11(12):1500. 10.3390/pathogens11121500 36558834 PMC9788015

[11] Jones JL Wang L Ceric O Nemser SM Rotstein DS Jurkovic DA Whole genome sequencing confirms source of pathogens associated with bacterial foodborne illness in pets fed raw pet food. J Vet Diagn Invest. 2019;31(2):235-40. 10.1177/1040638718823046 30663530 PMC6838835

[12] Hamame A Davoust B Cherak Z Rolain JM Diene SM . Mobile colistin resistance (mcr) genes in cats and dogs and their zoonotic transmission risks. Pathogens. 2022;11(6):698. 10.3390/pathogens11060698 35745552 PMC9230929

[13] Menezes J Moreira da Silva J Frosini SM Loeffler A Weese S Perreten V mcr-1 colistin resistance gene sharing between Escherichia coli from cohabiting dogs and humans, Lisbon, Portugal, 2018 to 2020. Euro Surveill. 2022;27(44):2101144. 10.2807/1560-7917.ES.2022.27.44.2101144 36330821 PMC9635019

[14] Nüesch-Inderbinen M Treier A Zurfluh K Stephan R . Raw meat-based diets for companion animals: a potential source of transmission of pathogenic and antimicrobial-resistant Enterobacteriaceae. R Soc Open Sci. 2019;6(10):191170. 10.1098/rsos.191170 31824726 PMC6837177

[15] Nilsson O . Hygiene quality and presence of ESBL-producing Escherichia coli in raw food diets for dogs. Infect Ecol Epidemiol. 2015;5(1):28758. 10.3402/iee.v5.28758 26490763 PMC4613903

[16] van Bree FPJ Bokken GCAM Mineur R Franssen F Opsteegh M van der Giessen JWB Zoonotic bacteria and parasites found in raw meat-based diets for cats and dogs. Vet Rec. 2018;182(2):50. 10.1136/vr.104535 29326391

[17] Finisterra L Duarte B Peixe L Novais C Freitas AR . Industrial dog food is a vehicle of multidrug-resistant enterococci carrying virulence genes often linked to human infections. Int J Food Microbiol. 2021;358(109284):109284. 10.1016/j.ijfoodmicro.2021.109284 34144837

[18] International Organization for Standardization (ISO). ISO 6579–1:2017. Microbiology of the food chain Horizontal method for the detection, enumeration and serotyping of Salmonella. Part 1: Detection of Salmonella spp. Geneva: ISO; 2022. Available from: https://www.iso.org/standard/56712.html 10.1016/j.ijfoodmicro.2018.03.022 29803313

[19] Mourão J Rebelo A Ribeiro S Peixe L Novais C Antunes P . Atypical non-H_2_S-producing monophasic Salmonella Typhimurium ST3478 strains from chicken meat at processing stage are adapted to diverse stresses. Pathogens. 2020;9(9):701. 10.3390/pathogens9090701 32859122 PMC7557518

[20] Ribeiro S Mourão J Novais Â Campos J Peixe L Antunes P . From farm to fork: Colistin voluntary withdrawal in Portuguese farms reflected in decreasing occurrence of mcr-1-carrying Enterobacteriaceae from chicken meat. Environ Microbiol. 2021;23(12):7563-77. 10.1111/1462-2920.15689 34327794

[21] Mourão J Ribeiro-Almeida M Novais C Magalhães M Rebelo A Ribeiro S From farm to fork: Persistence of clinically relevant multidrug-resistant and copper-tolerant Klebsiella pneumoniae long after colistin withdrawal in poultry production. Microbiol Spectr. 2023;11(4):e0138623. 10.1128/spectrum.01386-23 37428073 PMC10434174

[22] European Committee on Antimicrobial Susceptibility Testing (EUCAST). Recommendations for MIC determination of colistin (polymyxin E)—as recommended by the joint CLSI-EUCAST Polymyxin Breakpoints Working Group. Växjö: EUCAST. [Accessed: 5 Jan 2023]. Available from: https://www.eucast.org/fileadmin/src/media/PDFs/EUCAST_files/General_documents/Recommendations_for_MIC_determination_of_colistin_March_2016.pdf

[23] European Committee on Antimicrobial Susceptibility Testing (EUCAST). Breakpoint tables for interpretation of MICs and zone diameters, version 13.0. Växjö: EUCAST. [Accessed: 5 Jan 2022]. Available from: https://www.eucast.org/fileadmin/src/media/PDFs/EUCAST_files/Breakpoint_tables/v_13.0_Breakpoint_Tables.pdf

[24] Clinical and Laboratory Standards Institute (CLSI). M100. Performance standards for antimicrobial susceptibility testing. 32nd ed. Wayne: CLSI; 2022. Available from: https://clsi.org/media/wi0pmpke/m100ed32_sample.pdf

[25] Fujioka M Otomo Y Ahsan CR . A novel single-step multiplex polymerase chain reaction assay for the detection of diarrheagenic Escherichia coli. J Microbiol Methods. 2013;92(3):289-92. 10.1016/j.mimet.2012.12.010 23270615

[26] Mourão J Marçal S Ramos P Campos J Machado J Peixe L Tolerance to multiple metal stressors in emerging non-typhoidal MDR Salmonella serotypes: a relevant role for copper in anaerobic conditions. J Antimicrob Chemother. 2016;71(8):2147-57. 10.1093/jac/dkw120 27118781

[27] Bottari B Bancalari E Barera A Ghidini S Gatti M . Evaluating the presence of human pathogens in commercially frozen, biologically appropriate raw pet food sold in Italy. Vet Rec. 2020;187(7):e50. 10.1136/vr.105893 32430390

[28] Campos J Mourão J Peixe L Antunes P . Non-typhoidal Salmonella in the pig production chain: a comprehensive analysis of its impact on human health. Pathogens. 2019;8(1):19. 10.3390/pathogens8010019 30700039 PMC6470815

[29] European Food Safety Authority European Centre for Disease Prevention and Control . The European Union Summary Report on Antimicrobial Resistance in zoonotic and indicator bacteria from humans, animals and food in 2018/2019. EFSA J. 2021;19(4):e06490. 33868492 10.2903/j.efsa.2021.6490 PMC8040295

[30] Zeng H De Reu K Gabriël S Mattheus W De Zutter L Rasschaert G . Salmonella prevalence and persistence in industrialized poultry slaughterhouses. Poult Sci. 2021;100(4):100991. 10.1016/j.psj.2021.01.014 33610890 PMC7905466

[31] Finley R Ribble C Aramini J Vandermeer M Popa M Litman M The risk of salmonellae shedding by dogs fed Salmonella-contaminated commercial raw food diets. Can Vet J. 2007;48(1):69-75. 17310625 PMC1716752

[32] Groat EF Williams NJ Pinchbeck G Warner B Simpson A Schmidt VM . UK dogs eating raw meat diets have higher risk of Salmonella and antimicrobial-resistant Escherichia coli faecal carriage. J Small Anim Pract. 2022;63(6):435-41. 10.1111/jsap.13488 35191029 PMC9305152

[33] Centers for Disease Control and Prevention (CDC). Outbreak of multidrug-resistant Salmonella infections linked to contact with pig ear pet treats. Washington, D.C.: CDC; 2019. Available from: https://www.cdc.gov/salmonella/pet-treats-07-19/index.html

[34] Centers for Disease Control and Prevention (CDC). Multidrug-resistant salmonella infections linked to raw turkey products. Washington, D.C.: CDC; 2019. Available from: https://www.cdc.gov/salmonella/reading-07-18/index.html

[35] Bacci C Vismarra A Dander S Barilli E Superchi P . Occurrence and antimicrobial profile of bacterial pathogens in former foodstuff meat products used for pet diets. J Food Prot. 2019;82(2):316-24. 10.4315/0362-028X.JFP-18-352 30688534

[36] Campos J Gil J Mourão J Peixe L Antunes P . Ready-to-eat street-vended food as a potential vehicle of bacterial pathogens and antimicrobial resistance: An exploratory study in Porto region, Portugal. Int J Food Microbiol. 2015;206:1-6. 10.1016/j.ijfoodmicro.2015.04.016 25910073

[37] Hadjadj L Riziki T Zhu Y Li J Diene SM Rolain J-M . Study of mcr-1 gene-mediated colistin resistance in Enterobacteriaceae isolated from humans and animals in different countries. Genes (Basel). 2017;8(12):394. 10.3390/genes8120394 29257080 PMC5748712

[38] Bulochova V Evans EW . Exploring food safety perceptions and self-reported practices of pet owners providing raw meat–based diets to pets. J Food Prot. 2021;84(5):912-9. 10.4315/JFP-20-338 33428742

