# Supplementary material for: Raw meat-based diet for pets: a neglected source of human exposure to Salmonella and pathogenic Escherichia coli clones carrying mcr, Portugal, September 2019 to January 2020
Source: Euro Surveill. 2024 May 2;29(18):2300561. doi: 10.2807/1560-7917.ES.2024.29.18.2300561 (PMC11067432; doi:10.2807/1560-7917.ES.2024.29.18.2300561)
Supplement: Supplementary_Figure [file 23-00561_ANTUNES_SupplementaryFigure_S1.pdf]

Raw meat-based diet for pets: a neglected source of human exposure to *Salmonella* and pathogenic *Escherichia coli* clones carrying *mcr*, Portugal.

This supplementary material is hosted by Eurosurveillance as supporting information alongside the article "Raw meat-based diet for pets: a neglected source of human exposure to *Salmonella* and pathogenic *Escherichia coli* clones carrying *mcr*, Portugal", on behalf of the authors, who remain responsible for the accuracy and appropriateness of the content. The same standards for ethics, copyright, attributions and permissions as for the article apply. Supplements are not edited by Eurosurveillance and the journal is not responsible for the maintenance of any links or email addresses provided therein.

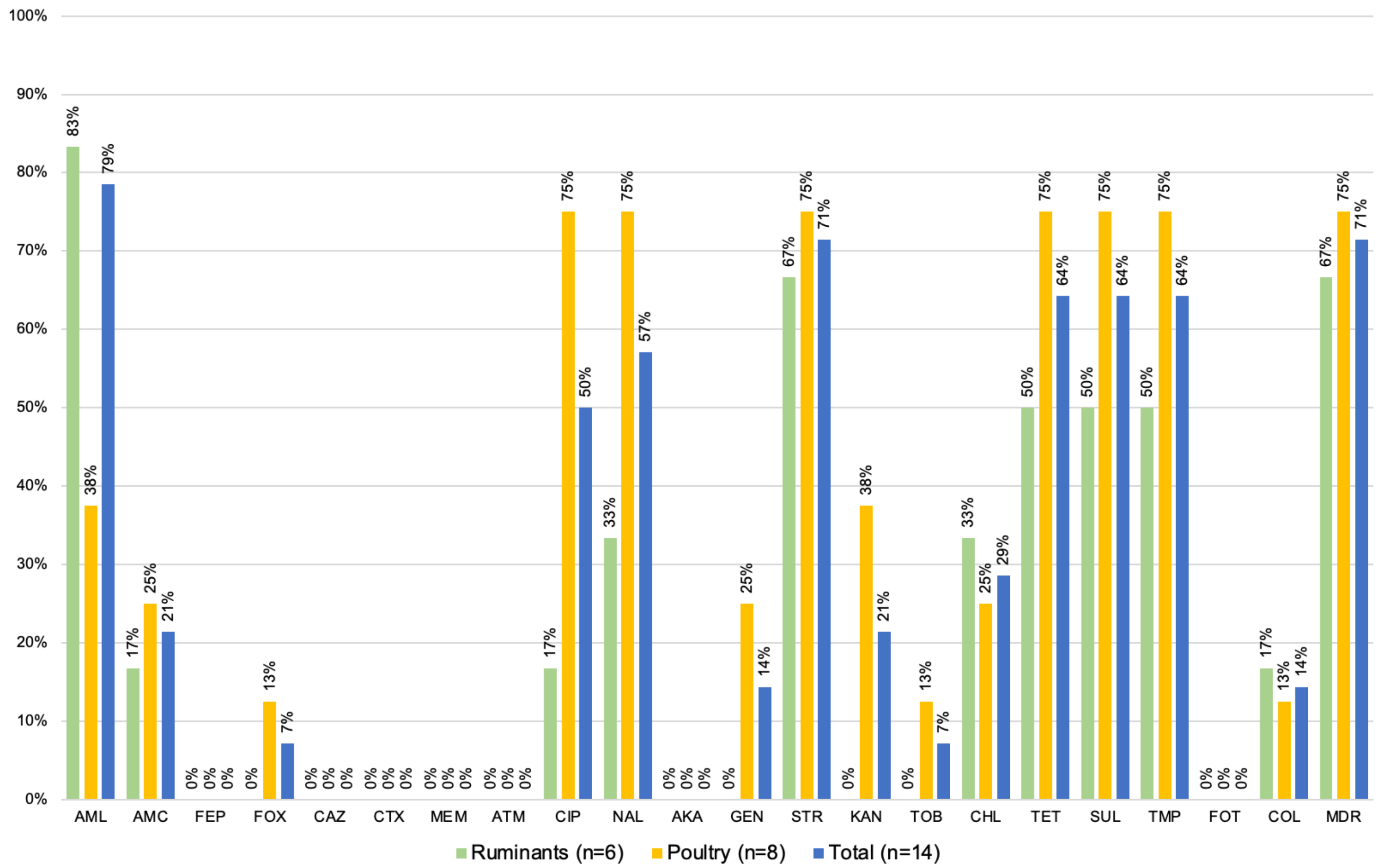

Fig. S1. Occurrence of antibiotic-resistant *E. coli* according to the food type. P>0,05 (Fisher exact test). Abbreviations: AML, amoxicillin; AMC, amoxicillin+clavulanic acid; FEP, cefepime; FOX, cefoxitin; CAZ, ceftazidime; CTX, cefotaxime; MEM, meropenem; ATM, aztreonam; CIP, ciprofloxacin; NAL, nalidixic acid; AKA, amikacin; GEN, gentamicin; STR, streptomycin; KAN, kanamycin; TOB, tobramycin; CHL, chloramphenicol; TET, tetracycline; SUL, sulphonamides; TMP, trimethoprim; FOT, fosfomycin; COL, colistin; MDR, multidrug resistance.
